# Influence of Supercritical Carbon Dioxide Extraction Conditions on Extraction Yield and Composition of *Nigella sativa* L. Seed Oil—Modelling, Optimization and Extraction Kinetics regarding Fatty Acid and Thymoquinone Content

**DOI:** 10.3390/molecules26216419

**Published:** 2021-10-24

**Authors:** Grzegorz Gawron, Wojciech Krzyczkowski, Robert Łyżeń, Leszek Kadziński, Bogdan Banecki

**Affiliations:** 1 Intercollegiate Faculty of Biotechnology of University of Gdansk and Medical University of Gdansk, University of Gdansk, Abrahama Str. 58, 80-307 Gdansk, Poland; grzegorz.gawron@phdstud.ug.edu.pl (G.G.); robert.lyzen@biotech.ug.edu.pl (R.Ł.); bogdan.banecki@biotech.ug.edu.pl (B.B.); 2 Biovico Sp. z o.o., Hutnicza Str. 15 B, 81-061 Gdynia, Poland; wojciech.krzyczkowski@biovico.pl

**Keywords:** *Nigella sativa*, thymoquinone, supercritical fluid extraction, response surface methodology, antibacterial activity

## Abstract

*Nigella sativa* L. is cultivated in many regions and its seeds have found use in variety of foods, but also in traditional medicine due to high content of biologically active essential oils. In this work optimization of supercritical carbon dioxide extraction from *N. sativa* seeds was performed using response surface methodology to describe the influence of extraction conditions on oil yield. Kinetics of oil and thymoquinone extraction were analyzed as well. It was demonstrated that in order to collect thymoquinone-rich *N. sativa* oil fraction, appropriate for health-related applications, the extraction should be carried out at 40 °C and 10–15 MPa. Following application of higher pressure of 35 MPa enables effective extraction of remaining oil rich in polyunsaturated fatty acids suitable for use in food industry. Thymoquinone-dependent antibacterial activity of the *N. sativa* seed oil was observed against bacterial pathogens: *Haemophilus influenzae, Staphylococcus haemolyticus, Staphylococcus epidermidis, Enterococcus faecalis* and *Escherichia coli*.

## 1. Introduction

**Figure 1:**
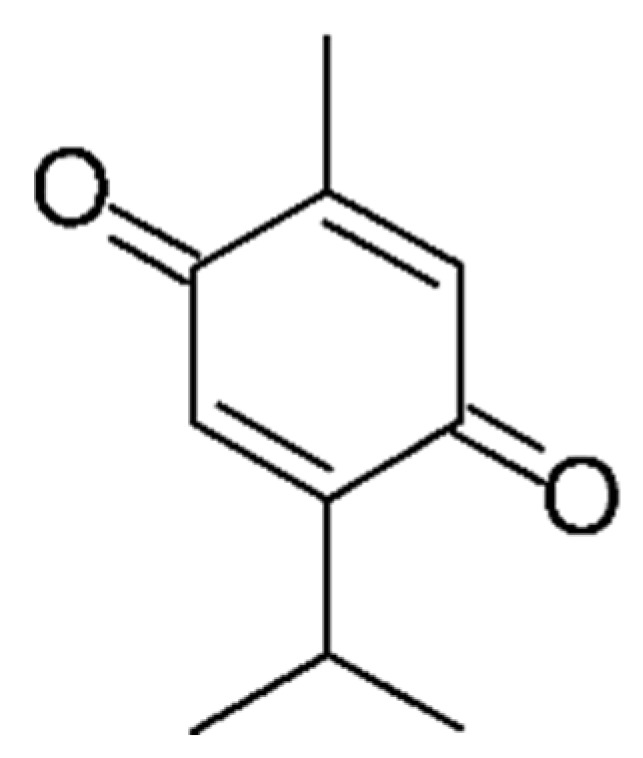
Chemical structure of thymoquinone.

*Nigella sativa* L. (NS), known as black cumin or black seed, belongs to *Ranunculaceae* family and is an annual herbaceous plant cultivated mainly in North-East Africa, Middle East, South-East Asia and also in some European countries. The nutritional value and characteristic taste of the NS seeds and seed oil make for their use as food ingredients in various cuisines as they constitute an abundant source of unsaturated fatty acids, essential oils, saponins, alkaloids, glycolipids, and fat-soluble vitamins [1,2,3]. Furthermore, the NS seeds are a rich source of biologically active compounds [4] and, in different forms, have been applied in traditional medicines of Middle and Far East to treat a vast variety of conditions, such as: hypertension, asthma, diabetes, inflammation, fever, kidney or liver disfunctions, cough, eczema or influenza [5,6,7]. The dominant contribution to the biological activity of NS seeds is attributed to thymoquinone (TQ) [4,5,8,9]. Some of its properties reported in recent studies are antioxidant [10,11], anti-inflammatory [7], anti-cancer [12,13], smooth muscle relaxant [14], antifungal [15] and antibacterial activity [16,17,18,19]. The chemical structure of thymoquinone is shown on Figure 1.

Various techniques have been implemented to extract biologically active compounds from plant material. Traditional methods include solvent extraction, Soxhlet extraction or steam distillation, while supercritical fluid extraction has been developed and applied in research as well as for industrial purposes more recently [20,21]. Supercritical fluids have density and solubility characteristic for a liquid, while the rate of diffusion, compressibility, viscosity and penetration rate are close to gases. This facilitates mass and heat transfer speed multiple times more efficient compared to the corresponding quantities for liquids, which results in a significant acceleration of the extraction processes. Supercritical CO_2_ is a Lewis base with proton-acceptor properties and polarity comparable to that of pentane or hexane This feature makes scCO_2_ an ideal candidate for extraction of essential oils which are mixture of non-polar, low-molecular compounds such as terpenes, aldehydes, ketones, alcohols, esters, phenols or lactones. The process takes place under anaerobic conditions, which protects the components against oxidation. CO_2_ is easily removed from the extract, it is non-flammable, non-corrosive, cheap, tasteless, colorless, inert and generally recognized as safe by Food and Drug Administration and European Food Safety Authority [22,23].

Supercritical carbon dioxide has turned out to be an alternative, green, eco-friendly solvent. Changing operative pressure and temperature cause significant changes in the density of the solvent and therefore it is possible to control the extract composition without the need of extent organic solvents consumption [24].

This study was designed and performed to determine scCO_2_ extraction conditions suitable for extraction of desired fractions of NS seed oil suitable for different industrial applications. Firstly, the NS seed oil extraction yield dependencies on scCO_2_ extraction conditions, i.e., temperature, pressure, scCO_2_ flow and extraction time and the yield of extraction from NS seeds were studied. Subsequently, the kinetics of thymoquinone and oil extraction at selected conditions was examined. As there are only few reports on the optimization of oil extraction from NS seeds using scCO_2_, which describe the effects of various extraction conditions on NS oil yield in the available literature [25,26,27], determination and mathematical description of the relationship between the parameters of the extraction process and the oil yield as well as TQ content in the oil presented in this work provides valuable information for parties interested in processing of NS seeds for food and medicinal purposes. The antibacterial activity of the NS seed oils differing in TQ concentration were examined against reference strains of human pathogens causing infections of skin, gastrointestinal tract and respiratory system: *Haemophilus influenzae*, *Staphylococcus haemolyticus*, *Staphylococcus epidermidis*, *Enterococcus faecalis*, *Escherichia coli*, *Shigella sonnei*, *Serratia odorifera* and *Salmonella typhimurium* and compared to effects of commercially available antiseptics, i.e., chlorquinaldol or a composition of amylmetacresol and 2,4-dichlorobenzyl alcohol to assess the oils’ potential as antibacterial agent.

## 2. Results

### 2.1. Modelling of Oil Extraction from Nigella sativa Seeds

Supercritical CO_2_ extraction of NS seeds under different conditions of pressure, temperature, extraction time and scCO_2_ flow gave oil yield from 6.13% to 31.15% (Table 1).

**Figure 2:**
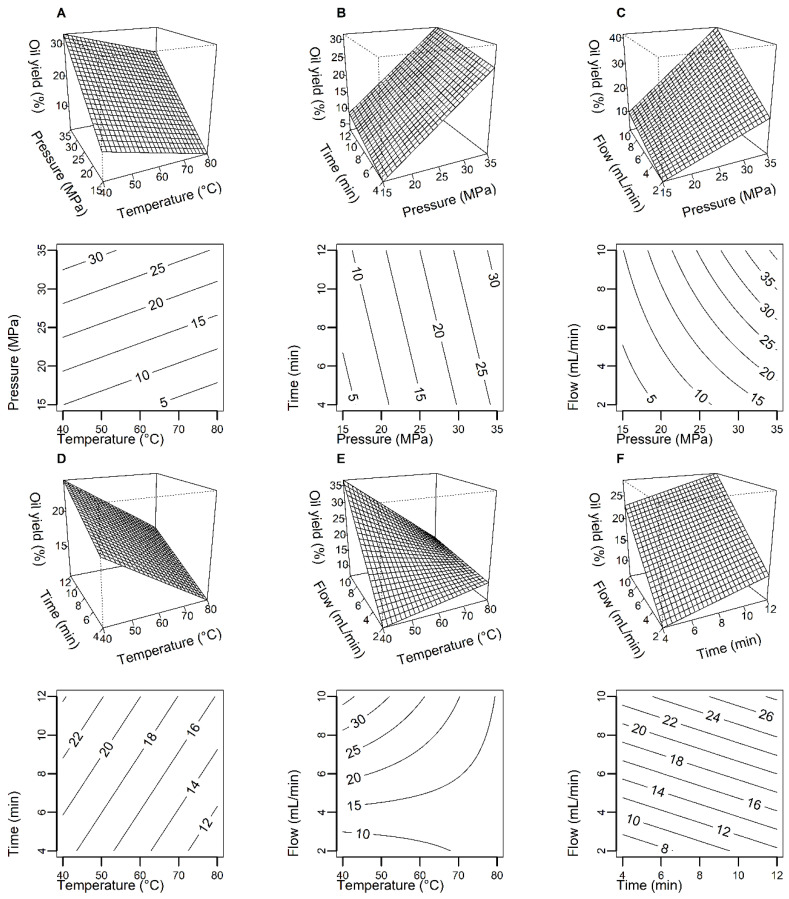
Response surface plots and corresponding contour plots showing the combined effect of pressure and temperature (**A**), extraction time and pressure (**B**), scCO_2_ flow and pressure (**C**), extraction time and temperature (**D**), scCO_2_ flow and temperature (**E**), scCO_2_ flow and extraction time on *N. sativa* oil yield (**F**). Other variables are fixed at their respective center point (pressure: 25 MPa, temperature: 60 °C, time: 8 min, scCO_2_ flow: 6 mL/min).

Statistical analysis of the CCRD 2^4^ model showed that the model is significant. All input variables studied have a statistically significant effect on the NS oil extraction efficiency. The highest impact on oil yield was exerted by pressure followed by scCO_2_ flow, temperature and time. Analysis of the response surface graphs (Figure 2) and the fitted model equation indicates that the lower the extraction temperature and the higher the pressure, the higher the extraction efficiency from NS seeds. Higher scCO_2_ flow and longer extraction time also increase the oil yield.

It was found that quadratic relations are insignificant, and the statistically significant interaction occurs between pressure and scCO_2_ flow as well as temperature and scCO_2_ flow (Table 2). This might be explained by the impact of both pressure and temperature have on scCO_2_ density, which can change from 225 g/L to 964 g/L at different conditions. Factors with negligible impact (*p* > 0.10) were removed from the model. The lack of fit of the model is insignificant. Values of adjusted and predicted coefficient of determination *R*^2^ are high and in good agreement and are 0.909 and 0.877, respectively.

The model has a high precision of 26.1 which indicates the adequate signal and can be used to predict the response. Equation of the model:

Yield (%) = −30.85 + 0.479·*P* + 0.303·*T*+ 0.679·*t* + 4.44·*F* + 0.110·p·*F* − 0.085·*T*·*F* (1)

where *P* is pressure (MPa), *T* is temperature (°C), *t* is time (min) and *F* is scCO_2_ flow (mL/min).

### 2.2. Kinetics of Extraction

**Figure 3:**
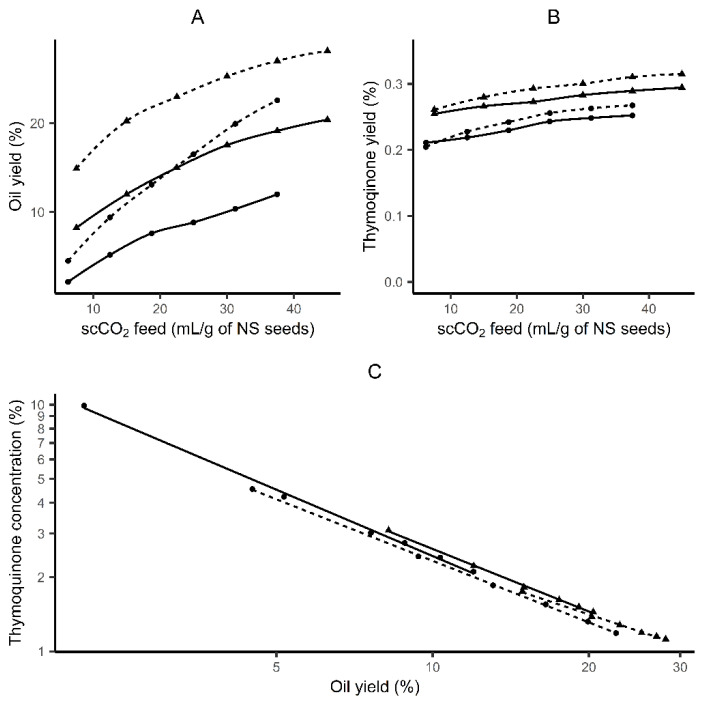
Relationship between NS seed oil yield and scCO_2_ feed expressed in mL per g of NS seeds obtained by scCO_2_ extraction at temperature of 40 °C, pressure of 10 (—) and 15 MPa (- - -) scCO_2_ flow of 5 (•) and 10 mL/min (▲) (**A**). Relationship between thymoquinone extraction yield and scCO_2_ feed expressed in mL per g of NS seeds obtained by scCO_2_ extraction at fixed temperature of 40 °C, pressure of 10 (—) and 15 MPa (- - -) and scCO_2_ flow of 5 (•) and 10 mL/min (▲) (**B**). Relationship between *N. sativa* oil yield and thymoquinone concentration (%, *w*/*w*) in oil during supercritical CO_2_ extraction at temperature of 40 °C, pressure of 10 (—) and 15 MPa (- - -) and scCO_2_ flow of 5 (•) and 10 mL/min (▲) (**C**). Mean values for three replicates were plotted.

The extraction curves (Figure 3) were obtained at 40 °C which was found favorable for essential oils extraction and combinations of pressure and scCO_2_ flow (10 or 15 MPa and 5 or 10 mL/min, respectively). The asymptotic yield of oil was ca. 28% (*w*/*w*). It was found that mass transfer of NS oil at 10 and 15 MPa is low and the period of constant extraction rate is extended, which allows fractionation of oil from NS (Figure 3).

A significant correlation between TQ content in NS oil and the oil yield was determined and described by logarithmic and power equations (Table 3). These observations contribute to better control of the extraction process to obtain the oil with the desired TQ content. The results obtained indicate that in order to isolate oil with high TQ content from the NS seeds, the process should be carried out at a low temperature of 40 °C and pressure of 10–15 MPa. Under these conditions, solubility in scCO_2_ is higher for essential oils than for the fixed oil. Thus, by controlling the amount of scCO_2_, i.e., extraction time, a given amount of oil can be obtained with the desired concentration of TQ.

### 2.3. Composition of Nigella sativa Seed Oil Fatty Acids

The results of determination of fatty acid composition of NS oils extracted using conditions employed in the CCRD experiments are presented in Table 4. Different conditions of the extraction haven’t significantly altered the contribution of each measured fatty acid to the total of fatty acids content in the NS oil obtained according to the results of ANOVA performed on the data (data analysis not shown). Linoleic acid was the most abundant of the fatty acids constituting 60.3 (SD = 1.11)%, followed by oleic acid, 22.7 (SD = 0.76)%, palmitic acid, 12.0 (SD = 0.65)%, stearic acid, 2.4 (SD = 0.13)%, eicosadienoic acid, 2.4 (SD = 0.16)% and myristic acid, 0.2 (SD = 0.03)%.

### 2.4. Antibacterial Activity of Nigella sativa Seed Extracts

Results of determination of selected human bacterial pathogens susceptibility to NSE1, NSE2, TQ, AMC/DCBA and CHQ are collected in Table 5. The strains determined as the most susceptible to TQ and NS seed oils were *Haemophilus influenzae* ATCC 43065, *Staphylococcus haemolyticus* ATCC 29970, *Staphylococcus epidermidis* ATCC 14990 and *Enterococcus faecalis* ATCC 19433 and with TQ MIC and MBC ranging from 4 to 16 µg/mL and 8 to 32 µg/mL, respectively. The antibacterial effectiveness of NS seed oils used in the experiments against the susceptible strains corresponded with their thymoquinone content. The remaining strains examined were less susceptible to TQ and NS seed oil with MIC of TQ ≥ 128 µg/mL, MIC of NSE1 ≥ 1.28 mg/mL and MIC of NSE2 ≥ 6.40 mg/mL. For most of the susceptible strains the MBC value of TQ and the NS seed oils were observed at respective MIC or 2 × MIC.

## 3. Discussion

Supercritical carbon dioxide extraction has been applied by researchers to collect biologically active extracts from NS seeds as this technique is considered reliable, efficient, provides space for adjustments to the extraction conditions and produces solvent-free products. The reports available in the literature concerning scCO_2_ extracts from NS seeds focus on chemical or biological properties of obtained extracts and present specific conditions at which the extracts were collected. The extracts properties and composition as well as the extraction yield varied among the studies due to different plant material and extraction conditions [19,28,29]. The studies presented in this work provide practical and comprehensive understanding of the influence of scCO_2_ extraction conditions on the extraction yield and demonstrate applicability of RSM and CCDR in as the framework for optimization of parameters for extraction of oils from NS seeds. Such an approach is a cost- and time-effective alternative to full factorial designs, which require a greater number of trials and has been applied by Salea et al. [26] in their work demonstrating applicability of Taguchi design as a mean for economical optimization of the scCO_2_ extraction from NS seeds. The presented kinetics of the oil and TQ extraction at low temperature and pressure exhibit a possibility of convenient and efficient separation of the TQ-rich fractions of the NS seed oil, while not compromising the efficiency of the overall TQ extraction. Kinetics studies of scCO_2_ extraction from NS seeds haven’t been reported in the available literature, however the results presented in this work are in agreement with kinetics of TQ and waxes extraction from aerial parts *Monarda didyma* and *M*. *fistulosa* studied by Sovova et al. [30]. The composition of fatty acids present in the oils extracted in this study hasn’t been shown to be significantly affected by the extraction conditions and was found to be typical for NS seed oil [1,27,28].

Supercritical fluids are characterized by viscosity similar to gases, density to liquids and high diffusivity. They have a variable solubility force that depends on their pressure and temperature. The lower the pressure and the higher the temperature, the scCO_2_ has lower density, and also a higher diffusion coefficient and permeability. On the other hand, too high diffusion coefficient preclude fractionation because compounds with different solubilities (i.e., essential oil and fatty acids glycerol esters) mix easily. In addition, the high temperature also lowers the viscosity of the fixed oil in seeds. The results obtained in this work indicate that the key to isolating the fraction with the highest concentration of thymoquinone with scCO_2_ is the use of the low temperature to maintain a moderate scCO_2_ density (578 g/mL and 750 g/mL at 40 °C and the pressure of 10 MPa and 15 MPa, respectively) and low flow of scCO_2_ which allows to capture the right mass or volume of extract in a separator when collecting the thymoquinone-rich fraction.

The antibacterial activity of NS seed oil and TQ shown in this work remain in agreement with results of previous research on the topic. The NS seed extracts obtained with use of various techniques as well as TQ have been demonstrated to have a strong bactericidal effect against *S. aureus* and *S. epidermidis* [17,28,31,32,33]. The antibacterial effects of NS seed oil and TQ against *H. influenzae* and *S. haemolyticus* shown in this work hasn’t been previously demonstrated and suggest applicability of the oil and TQ against a broad spectrum of bacterial pathogens. The results concerning NS seed extracts and TQ efficiency against *E. coli*, *E. faecalis* shown in this study and present in the available literature might indicate on strain-dependency of susceptibility these bacteria to the tested substances [16,19,28,32]. The antibacterial effects of NS seed oil were observed to be strongly dependent on the final TQ concentration in the samples.

## 4. Materials and Methods

### 4.1. Optimization of Supercritical Carbon Dioxide Extraction from Nigella sativa Seeds

#### 4.1.1. Plant Material and Extraction Equipment

**Figure 4:**
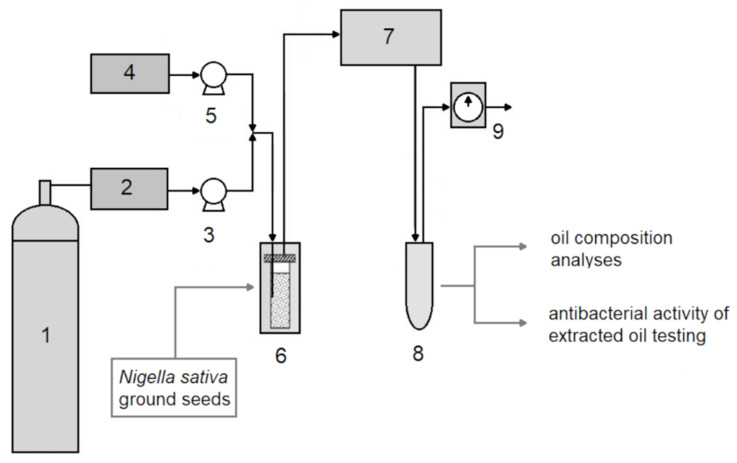
Flow diagram of a supercritical carbon dioxide extraction system (1. CO_2_ supply; 2. cooling heat exchanger; 3. CO_2_ pump; 4. co-solvent vessel; 5. co-solvent pump; 6. extraction oven with extraction vessels; 7. backpressure regulator; 8. sample collection; 9. wet gas meter and CO_2_ release).

*Nigella sativa* L. seeds were purchased from FZL company from Poland, batch number F4568. The Voucher specimen No WLDK/2013 is stored at Biovico, Gdynia, Poland. The seeds were ground with a Bosch KM13 grinder (Bosch, Munich, Germany), 10 g at once for 2 min. Ground seeds (4.0 g) were put in 10 mL stainless steel extraction vessels. Supercritical CO_2_ extraction of NS seed oil was performed with a Waters MV-10 ASFE (Waters Corporation, Milford, MA, USA) extractor at different conditions of temperature, pressure, extraction time, and scCO_2_ flow (Figure 4). CO_2_ of 99.995% purity (AirLiquide, Cracow, Poland) was used for extraction of NS oil.

#### 4.1.2. Modelling of Oil Extraction from the *Nigella sativa* Seeds

The analysis of the effects of pressure, temperature, extraction time and scCO_2_ flow on the oil extraction efficiency from NS seeds was performed using a central composite rotatable design (CCRD) with response surface methodology (RSM). The experimental design used in this study was a 2^4^ factorial CCRD. Levels of independent variables are shown in Table 6 while the experimental matrix is shown in Table 1.

The relationship of the extraction parameters (independent variables) and NS oil yield (the response) was fitted to a predictive second order polynomial equation:

(2) $$Y_{i}=\beta_{0}+\sum_{i=1}^{n}\beta_{i}X_{i}+\sum_{i=1}^{n}\beta_{ii}X_{i}^{2}+\sum_{i=1}^{n-1}\sum_{j=i+1}^{n}\beta_{ij}X_{i}X_{j})$$

where *Y_i_* is the predicted response (NS oil yield), subscripts *i* and *j* have values from 1 to the number of variables; *β*_0_ is a constant; *β*_*i*_, linear coefficient; *β*_*ii*_, the quadratic coefficient; *β*_*ij*_, cross-product coefficient; *n*, number of factors and *X*_*i*_ and *X*_*j*_ are the coded dimensionless values of the analyzed variables. The software Design-Expert 7 (Stat-Ease, Minneapolis, MN, USA) was used for experimental design, the analysis of variance (ANOVA), and graphical analysis of the data. The statistical significance of the model was expressed by an *F*-test and the quality of its fit was evaluated by the coefficient of determination *R*^2^. The significance of the regression coefficients was tested by the Student’s *t*-test and the *p* values were used to determine the significance of each coefficient.

#### 4.1.3. Kinetics of Thymoquinone and Oil Extraction

In order to investigate the kinetics of extraction of TQ and oil from NS seeds, extraction was carried out in different operating pressure of 10 and 15 MPa, scCO_2_ flow of 5 and 10 mL/min and extraction time from 3 to 24 min. Those conditions were chosen basing on the results of modelling of oil extraction from the NS seeds. All extractions were triplicated. The extracts were accurately weighted and submitted to GC-MS analysis in order to establish thymoquinone content.

### 4.2. Oil Composition Analyses

#### 4.2.1. Gas Chromatography-Mass Spectrometry Analysis (GC-MS)

Chemical composition of oils obtained by scCO_2_ extraction was analyzed using PerkinElmer^®^ Clarus^®^ 500 (PerkinElmer, Waltham, MA, USA) gas chromatograph equipped with PerkinElmer^®^ Clarus^®^ 500 mass spectrometer and controlled by PerkinElmer^®^ TurboMass^TM^ GC/MS software v. 5.0 using NIST spectral libraries v. 2.0.0. PerkinElmer^®^ Elite-5MS (30 m × 0.25 mm, 0.25 µm) was the column used for these analyses. Prior to the analyses the 20 µL of each oil sample was diluted in 380 µL of isooctane. The injection volume was 1 µL in split mode 1:10, injector temperature was 250 °C, flow rate was 1.5 mL/min and the electron ionization energy was 70 eV. Initial oven temperature of 40 °C was increased to 200 °C after 5 min at a rate of 10 °C/min, then increased to 280 °C at a rate of 5 °C/min and held for 8 min. Standards and solvent were obtained from Sigma-Aldrich, Poznan, Poland. Each analysis was made in triplicate.

#### 4.2.2. Gas Chromatography Fatty Acid Analysis

Composition of fatty acids in extracted NS seed oils was analyzed using PerkinElmer^®^ Clarus^®^ 500 gas chromatograph with flame-ionization detector controlled by PerkinElmer^®^ TotalChrom^TM^ software v. 6.3.2 and the column used was PerkinElmer^®^ Elite-5MS (30 m × 0.25 mm, 0.25 µm). Prior to the analysis 20 µL of oil was dissolved in 0.4 mL of 0.5 M KOH in methanol and incubated for 5 min in 100 °C to hydrolyze the triacylglycerols, then 0.35 mL of 20% BF_3_ in methanol was added and samples were incubated for 5 min in 100 °C to produce fatty acid methyl esters (FAME). After cooling to room temperature 0.5 mL of saturated NaCl water solution and 0.5 mL of isooctane were added and samples were vortex-mixed. Isooctane fraction was collected for GC analysis. Standard solutions of methyl myristate, methyl palmitate, methyl linoleate, methyl oleate, methyl stearate and methyl eicosadienoate were prepared in isooctane. Injection volume was 0.5 µL in split mode 1:10, injector temperature was 250 °C, flow rate was 1.5 mL/min and FID temperature was 250 °C. Initial oven temperature of 180 °C was increased to 195 °C after 2 min at a rate of 1 °C/min, then increased to 250 °C at a rate of 7 °C/min and held for 5 min. The FAME standards applied were obtained from Sigma-Aldrich. Analyzes were made in triplicates.

### 4.3. Antibacterial Activity of Nigella sativa Seed Oil

#### 4.3.1. Bacterial Strains

Eight human bacterial pathogens were represented by reference strains: *Staphylococcus haemolyticus* ATCC 29970, *Staphylococcus epidermidis* ATCC 14990, *Enterococcus faecalis* ATCC 19433, *Escherichia coli* ATCC 25922, *Haemophilus influenzae* ATCC 43065, *Salmonella typhimurium* ATCC 13311, *Serratia odorifera* ATCC 33077 and *Shigella sonnei* ATCC 9290.

#### 4.3.2. Preparation of *Nigella sativa* Seed Oils for Evaluation of Antibacterial Activity

Freshly ground NS seeds were extracted with scCO_2_ at 40 °C and pressure of 10 MPa, flow 5 mL/min for 10 min to obtain NSE1 oil with high concentration of TQ (9.91%). The oil fraction NSE2 containing lower TQ concentration (2.10%) was obtained by prolongation of the extraction at these conditions to total of 30 min.

#### 4.3.3. Minimum Inhibitory Concentration Determinations

Minimum inhibitory concentrations (MIC) were determined with use of a broth microdilution protocol following Clinical and Laboratory Standards Institute guidelines [30]. Stock solutions of TQ, chlorquinaldol (CHQ) and NS oils were prepared in dimethyl sulfoxide (DMSO), while the stock solution of a composition of amylmetacresol with 2,4-dichlorobenzyl alcohol in 1:2 molar ratio (AMC/DCBA) was prepared in ethanol. Maximum final concentrations were 512 µg/mL for TQ and CHQ, 512 µg/mL of AMC with 1024 µg/mL of DCBA in AMC/DCBA samples. Two variants of *N. sativa* oil, NSE1 and NSE2 (9.91% and 2.10% of TQ, respectively) were used at maximum final concentrations of 5.12 mg/mL and 25.6 mg/mL. Two-fold serial dilutions in Mueller-Hinton broth (MHB) were prepared on 96-well plates. DMSO and ethanol were used as controls. Overnight bacterial cultures were diluted in fresh MHB medium and cultured to obtain log phase, then bacterial suspensions of 10^6^ CFU/mL in MHB were prepared and used to inoculate the 96-well plates. Plates were then incubated for 24 h in 37 °C in sealed plates to prevent TQ evaporation. Standards of antibacterial agents, MHB and solvents were obtained from Sigma-Aldrich.

#### 4.3.4. Minimum Bactericidal Concentration Determinations

Minimum bactericidal concentrations (MBC) were determined by sub-culturing samples collected from the wells of microtiter plates after the broth microdilution assay performed to determine MIC values for tested substances. Samples from the wells showing no visible growth were serially diluted in sterile 0.85% NaCl and 10 µL of each sample was spread on Mueller-Hinton agar plates. After 24 h incubation at 37 °C, the colonies were counted and results were compared to control. Minimum concentration reducing viable cell count by ≥ 99.9% was considered as MBC.

### 4.4. Statistical Analyses

All experiments were performed in three or more repetitions. Statistical analyses of the extraction models were performed using Design-Expert 7 (Stat-Ease, Minneapolis, MN, USA). Differences between means were analyzed with the Student’s *t*-test for independent samples with Microsoft Excel Data Analysis Tool Pack and considered significant if *p* value was below 0.05.

## 5. Conclusions

In the course of presented work influence of scCO_2_ extraction conditions, i.e., scCO_2_ flow rate, temperature, pressure and extraction time on extraction yield of *N. sativa* seed oil, as well as extraction kinetics regarding total oil and thymoquinone were defined. The dependencies were expressed with a reliable mathematical model, which facilitates planning of extraction towards obtaining oil of desired final composition, especially thymoquinone-rich fractions for use in cosmetic and curative formulations and fixed oil fraction appropriate for use in food industry as the fatty acid composition was not significantly affected by extraction conditions. The optimized scCO_2_ extraction was used to collect thymoquinone-rich *N. sativa* seed oils. Examination of antibacterial activity of the oils substantiate the importance of separation of thymoquinone-rich *N. sativa* oil fractions as their bactericidal activity against pathogenic bacteria, which might contribute to establishment of novel efficient means of preventing bacterial infections, was demonstrated.

## Figures and Tables

**Table 1:** Experimental matrix and results in central composite rotatable design 2^4^ experiment; *X*_1_—pressure (MPa), *X*_2_—temperature (°C), *X*_3_—extraction time (min), *X*_4_—scCO_2_ flow (mL/min). Coded values of the variables are in parentheses.

No	*X*_1_, Pressure (MPa)	*X*_2_, Temperature (°C)	*X*_3_, Time (min)	*X*_4_, scCO_2_ Flow (mL/min)	NS Oil Yield (%)	
					Actual	Predicted
1	20 (−1)	50 (−1)	6 (−1)	4 (−1)	7.03	7.51
2	20 (−1)	50 (−1)	6 (−1)	8 (1)	16.47	17.07
3	20 (−1)	50 (−1)	10 (1)	4 (−1)	9.58	10.23
4	20 (−1)	50 (−1)	10 (1)	8 (1)	18.63	19.79
5	20 (−1)	70 (1)	6 (−1)	4 (−1)	6.79	6.77
6	20 (−1)	70 (1)	6 (−1)	8 (1)	7.62	9.53
7	20 (−1)	70 (1)	10 (1)	4 (−1)	7.65	9.49
8	20 (−1)	70 (1)	10 (1)	8 (1)	8.71	12.25
9	30 (1)	50 (−1)	6 (−1)	4 (−1)	15.95	16.70
10	30 (1)	50 (−1)	6 (−1)	8 (1)	31.15	30.66
11	30 (1)	50 (−1)	10 (1)	4 (−1)	20.91	19.42
12	30 (1)	50 (−1)	10 (1)	8 (1)	30.05	33.38
13	30 (1)	70 (1)	6 (−1)	4 (−1)	17.13	15.96
14	30 (1)	70 (1)	6 (−1)	8 (1)	23.59	23.12
15	30 (1)	70 (1)	10 (1)	4 (−1)	17.65	18.68
16	30 (1)	70 (1)	10 (1)	8 (1)	24.89	25.84
17	15 (-α)	60 (0)	8 (0)	6 (0)	10.62	5.89
18	35 (α)	60 (0)	8 (0)	6 (0)	29.69	28.67
19	25 (0)	40 (-α)	8 (0)	6 (0)	22.07	21.42
20	25 (0)	80 (α)	8 (0)	6 (0)	15.05	13.14
21	25 (0)	60 (0)	4 (-α)	6 (0)	11.22	14.56
22	25 (0)	60 (0)	12 (α)	6 (0)	21.35	19.99
23	25 (0)	60 (0)	8 (0)	2 (-α)	6.13	8.92
24	25 (0)	60 (0)	8 (0)	10 (α)	27.08	25.64
25	25 (0)	60 (0)	8 (0)	6 (0)	17.71	17.28
26	25 (0)	60 (0)	8 (0)	6 (0)	18.54	17.28
27	25 (0)	60 (0)	8 (0)	6 (0)	18.68	17.28
28	25 (0)	60 (0)	8 (0)	6 (0)	17.55	17.28
29	25 (0)	60 (0)	8 (0)	6 (0)	21.15	17.28
30	25 (0)	60 (0)	8 (0)	6 (0)	18.44	17.28

**Table 2:** Analysis of variance (ANOVA) for central composite rotatable design 2^4^.

Source	SS	df	MS	*F*	*p*
Model	1415	6	226	49.2	<0.0001
A—scCO_2_ pressure	782	1	782	163	<0.0001
B—scCO_2_ temperature	103	1	103	21.6	<0.0001
C—Extraction time	44.3	1	44.3	9.25	0.0058
D—scCO_2_ flow	419	1	419	87.6	<0.0001
AD	19.5	1	19.5	4.07	0.0555
BD	46.4	1	46.4	9.68	0.0049
Residual	110	23	4.79		
Lack of Fit	102	18	5.65	3.37	0.0918
Pure Error	8.40	5	1.68		
Cor Total	1525	29			

**Table 3:** Equations describing the relationship between *N. sativa* oil yield (*X*) and thymoquinone content in oil (*Y*) in different conditions of pressure and scCO_2_ flow during extraction at 40 °C.

Pressure (MPa)	scCO_2_ Flow (mL/min)	Logarithmic Equation	Power Equation	*R* ^2^
10	5	ln*Y* = −0.893·ln*X* + 2.974	*Y* = 19.0·*X*^−0.893^	0.9979
10	10	ln*Y* = −0.843·ln*X* + 2.896	*Y* = 18.1·*X*^−0.843^	0.9987
15	5	ln*Y* = −0.829·ln*X* + 2.754	*Y* = 15.7·*X*^−0.829^	0.9997
15	10	ln*Y* = −0.704·ln*X* + 2.454	*Y* = 11.6·*X*^−0.704^	0.9972

**Table 4:** Contributions of myristic (C:14), palmitic (C16:0), linoleic (C18:2), oleic (C18:1), stearic (C18:0) and eicosadienoic acids (C20:2) in the total of fatty acids in the NS seed oils obtained at different conditions of scCO_2_ pressure, scCO_2_ temperature, extraction time and flow of scCO_2_ (results of FAME analysis by GC-FID).

Pressure (MPa)	Temperature (°C)	Time (min)	scCO_2_ Flow (mL/min)	C14:0 (%)	C16:0 (%)	C18:2 (%)	C18:1 (%)	C18:0 (%)	C20:2 (%)
20	50	6	4	0.2	11.4	60.9	22.6	2.4	2.5
20	50	6	8	0.2	12.5	60.8	21.8	2.4	2.3
20	50	10	4	0.2	12.6	61.3	21.5	2.3	2.0
20	50	10	8	0.2	12.2	60.6	22.2	2.4	2.4
20	70	6	4	0.3	13.8	57.4	23.8	2.7	2.1
20	70	6	8	0.3	13.3	58.0	24.0	2.4	2.0
20	70	10	4	0.2	12.4	57.5	25.2	2.5	2.2
20	70	10	8	0.2	13.2	60.1	21.8	2.4	2.2
30	50	6	4	0.2	11.4	61.3	22.3	2.3	2.5
30	50	6	8	0.2	11.4	61.1	22.5	2.3	2.5
30	50	10	4	0.2	11.9	60.7	22.3	2.5	2.5
30	50	10	8	0.2	11.9	60.6	22.3	2.5	2.5
30	70	6	4	0.2	11.5	60.1	23.4	2.4	2.4
30	70	6	8	0.2	11.8	59.8	23.2	2.5	2.5
30	70	10	4	0.2	11.6	59.7	23.7	2.4	2.3
30	70	10	8	0.2	11.7	60.1	23.1	2.5	2.4
15	60	8	6	0.2	11.7	61.2	22.5	2.2	2.2
35	60	8	6	0.2	11.9	60.6	22.3	2.4	2.5
25	40	8	6	0.2	10.6	62.3	22.3	2.1	2.5
25	80	8	6	0.2	12.0	60.8	22.3	2.3	2.3
25	60	4	6	0.3	11.4	60.6	22.8	2.5	2.5
25	60	12	6	0.2	12.0	60.9	22.2	2.3	2.4
25	60	8	2	0.2	11.4	61.1	22.5	2.3	2.4
25	60	8	10	0.2	12.5	58.7	23.3	2.7	2.6
25	60	8	6	0.2	11.9	60.7	22.4	2.4	2.5

**Table 5:** Minimal inhibitory concentrations (MIC) or minimal bactericidal concentrations (MBC) of thymoquinone (TQ), thymol (THY), *p*-cymene (CY), *Nigella sativa* seed extracts obtained by supercritical CO_2_, extraction (NSE1 and NSE2), chlorquinaldol (CHQ) and a combination of amylmetacresol and 2,4-dichlorobenzyl alcohol (AMC/DCBA) in a 1:2 molar ratio against chosen pathogenic bacteria. For AMC/DCBA combination the concentration of amylmetacresol was presented.

Strain	NSE1 (mg/mL)		NSE2 (mg/mL)		TQ (µg/mL)		AMC/DCBA (µg/mL)		CHQ (µg/mL)	
	MIC	MBC	MIC	MBC	MIC	MBC	MIC	MBC	MIC	MBC
*H. influenzae* ATCC 43065	0.04	0.08	0.20	0.40	4	8	128	128	4	16
*S. haemolyticus* ATCC 29970	0.08	0.08	0.40	0.40	8	8	128	128	4	32
*S. epidermidis* ATCC 14990	0.08	0.32	0.4	1.6	8	32	32	32	16	64
*E. faecalis* ATCC 19433	0.16	0.32	0.80	1.60	16	32	64	128	8	16
*E. coli* ATCC 25922	1.28	1.28	6.40	6.40	128	128	256	512	16	64
*S. sonnei* ATCC 9290	2.56	>5.12	12.80	>25.6	256	>512	128	256	16	64
*S. odorifera* ATCC 33077	5.12	>5.12	25.60	>25.6	512	>512	128	512	64	64
*S. typhimurium* ATCC 13311	5.12	>5.12	25.60	>25.6	512	>512	64	128	32	64

**Table 6:** Levels of variables in central composite rotatable design 2^4^ experiment.

Independent Variables	Levels				
	−α	−1	0	1	α
*X*_1_—scCO_2_ pressure (MPa)	15	20	25	30	35
*X*_2_—scCO_2_ temperature (°C)	40	50	60	70	80
*X*_3_—Extraction time (min)	4	6	8	10	12
*X*_4_—scCO_2_ flow (mL/min)	2	4	6	8	10

## Data Availability

Not applicable.

## Sample Availability

Not available.
